# Modulation of Intestinal Immune and Barrier Functions by Vitamin A: Implications for Current Understanding of Malnutrition and Enteric Infections in Children

**DOI:** 10.3390/nu10091128

**Published:** 2018-08-21

**Authors:** Pedro Henrique Q. S. de Medeiros, Daniel V. Pinto, Juliana Zani de Almeida, Juliana M. C. Rêgo, Francisco A. P. Rodrigues, Aldo Ângelo M. Lima, David T. Bolick, Richard L. Guerrant, Reinaldo B. Oriá

**Affiliations:** 1 Laboratory of Infectious Diseases, Institute of Biomedicine, School of Medicine, Federal University of Ceara, Fortaleza 60430-270 CE, Brazil; phquintela@hotmail.com (P.H.Q.S.d.M.); delvanefapr@gmail.com (F.A.P.R.); alima@ufc.br (A.Â.M.L.); 2 Division of Infectious Diseases and International Health, Department of Medicine, University of Virginia School of Medicine, Charlottesville, VA 22908, USA; dtb5w@virginia.edu (D.T.B.); rlg9a@virginia.edu (R.L.G.); 3 Laboratory of Tissue Healing, Ontogeny and Nutrition, Department of Morphology and the Institute of Biomedicine, School of Medicine, Federal University of Ceara, Fortaleza 60430-270 CE, Brazil; danielvieirapinto@gmail.com (D.V.P.); juliana_zani@yahoo.com.br (J.Z.d.A.); nutrijulianarego@gmail.com (J.M.C.R.); 4 Department of Nutrition, Christus University Center, Fortaleza 60190-060 CE, Brazil

**Keywords:** vitamin A supplementation, vitamin A deficiency, intestinal immune response, intestinal barrier function, enteric infections

## Abstract

The micronutrient vitamin A refers to a group of compounds with pleiotropic effects on human health. These molecules can modulate biological functions, including development, vision, and regulation of the intestinal barrier. The consequences of vitamin A deficiency and supplementation in children from developing countries have been explored for several years. These children live in an environment that is highly contaminated by enteropathogens, which can, in turn, influence vitamin A status. Vitamin A has been described to modulate gene expression, differentiation and function of diverse immune cells; however, the underlying mechanisms are not fully elucidated. This review aims to summarize the most updated advances on elucidating the vitamin A effects targeting intestinal immune and barrier functions, which may help in further understanding the burdens of malnutrition and enteric infections in children. Specifically, by covering both clinical and in vivo/in vitro data, we describe the effects of vitamin A related to gut immune tolerance/homeostasis, intestinal barrier integrity, and responses to enteropathogens in the context of the environmental enteric dysfunction. Some of the gaps in the literature that require further research are also highlighted.

## 1. Introduction

Vitamin A derivatives (or retinoids) are major nutrients for human health and modulate several functions, such as cell differentiation, proliferation, and apoptosis [1,2]. Retinoids are used for xerophthalmia and blindness prevention [3], and abnormal levels may be associated with teratogenic alterations [4,5]. Retinoids are also key micronutrients for improving malnutrition and enteric illnesses and related child mortality and morbidity (aged <5 years old) in endemic areas of the developing world, even being formerly called an “anti-infective agent” [6]. However, vitamin A beneficial effects may be dependent on pathogen-driven immune response [7] and genetic background [8]. Although much progress has been made on elucidating the vitamin A effects on intestinal barrier function, many gaps remain in our understanding of its interactions with the intestinal microbiota, mucosal immune system, and epithelial junctional proteins, which have been the subject of recent research. In this review, we summarize recent data on vitamin A effects on intestinal epithelial barrier proteins and mucosal barrier function and underlying immune responses both in preclinical and clinical studies of enteric infections and malnutrition.

## 2. Cellular and Molecular Mechanisms of Vitamin A in the Gut: Crosstalk of Immune and Inflammatory Responses 

Vitamin A is the term encompassing a group of fat-soluble compounds (retinol, retinal, and retinoic acid). It is an essential nutrient and the primary dietary sources of vitamin A consist of carotenoids (provitamin A from plants) and retinyl esters (preformed vitamin A from animal sources). The major examples of carotenoids are α-carotene, β-carotene, and β-cryptoxanthin, while retinylpalmitate is the most predominant ester [9,10]. Provitamin A carotenoids are cleaved into all-trans retinoic acid (RA), which is the most biologically active form of vitamin A [11]. The majority of retinyl esters are deposited in the liver (in stellated cells), where they are hydrolyzed to retinol and then bound to retinol-binding protein (RBP). The uptake of vitamin A from the RBP was recently described to rely on the expression of stimulated retinoic acid gene 6 (Stra6), which functions as a receptor for RBP [12]. All *trans*-retinoic acid binds to retinoic acid receptors (RARs) and retinoid X receptors (RXRs) from the nuclear hormone receptor family [13]. RAR/RXR heterodimers bind to consensus promoter DNA sequences, called retinoic acid response elements (RAREs), and they act as transcription factors for several genes.

The effective implementation of vitamin A policies to treat malnutrition and enteric diseases depends on better understanding of its biological functions. While the classical direct antioxidant action of vitamin A is well known, other effects targeting intestinal immune cells, intestinal epithelium, and microbiota have been described in the past few years. It has been recognized that several pathways are influenced by retinoids in the gut, mainly through gene expression modulation [14], with the regulation of several mediators of the immune system, including both pro-inflammatory and anti-inflammatory responses.

The intestine is considered an important site of antigenic interaction, since it is in constant interface with commensal, pathogenic microorganisms, as well as molecules derived from ingested foods. The intestinal immune system comprises both innate and adaptive factors, such as T and B lymphocytes (IgA secreting cells), dendritic cells (DC), macrophages, together with the commensal microbiota, mucus, and antimicrobial substances that were produced by intestinal cells, all that interact to provide the steady balance between physiological and pathogenic agents residing in this microenvironment [15]. 

Major advances during the past few years have shed light on the understanding of how retinoic acids regulate intestinal immune tolerance to commensal bacteria/food antigens [16,17] and anti-inflammatory responses by T-cell modulation [11]. CD103^+^-DC-derived RA has been shown to induce gut-homing receptors (α4β7-integrin and the chemokine receptor, CCR9) on T cells [18], which may improve the efficacy of oral vaccines [19]. In addition, RA derived from DCs can promote IL-10 producing-T cells [20], with the involvement of Toll-like receptors [21], functioning as suppressive Foxp3^+^ T regulatory (Treg) cells. The evidence of vitamin A mucosal protective effects involving T-lymphocytes (which may be important to control chronic inflammatory conditions, such as the T-cell driven-environmental enteric dysfunction (EED) in children) [22,23] has been accumulating. RA-preconditioned human Tregs displayed almost complete resistance to Th1 and Th17 conversion and sustain Foxp3 expression (suppressive function), following IL-1 and IL-6 stimulation [24]. In addition, the transfer of pretreated Treg cells with RA further enhances anti-inflammatory effects on xenograft-vs-host disease [24]. Indeed, the potential use of natural Treg cells primed with RA has been suggested for treatment of chronic immune-mediated diseases [25]. High concentrations of RA or vitamin D metabolites, as well as thymic stromal lymphopoietin or TGF-β, activate signaling programs in dendritic cells, which result in the priming of Treg cell responses [26]. Conversely, vitamin A deficiency may alter the intestinal microbiota and impair Th17 differentiation [27,28], which may compromise enteric pathogen clearance.

After being absorbed by the intestinal mucosa, the retinol is converted into retinaldehyde by retinol dehydrogenase (RDH). Retinal becomes RA by retinaldehyde dehydrogenase activity [29] in dendritic cells [30], but also in intestinal epithelial and a subset of lamina propria stromal cells [31,32]. In addition, RA can be degraded through the action of cytochrome P450 family 26 enzymes (CYP26) [27]. Dendritic cells secrete more TGF-β when stimulated by RA, favoring the Treg cell differentiation in the intestine under homeostasis [16,33]. TGF-β is a key cytokine regulating intestinal mucosal IgA secreting cells [34]. In addition, RA that is derived from gut associated lymphoid tissue (GALT)-DCs can induce mucosal IgA secretion in the small intestine under IL-6 or IL-5 modulation [35].

RA also binds to the retinoic acid receptors (RAR) in CD4^+^ T cell nucleus for upregulating α4β7 integrin expression, while activated RAR/RXR heterodimers bind to RARE for increasing expression of CCR9 on the lipid membrane surface of T-cells [36]. CCL25-induced lymphocyte adhesion to MadCAM-1 is mediated by α4β7-integrin in response to intestinal inflammation [37]. RA can also block the expression of RORγt nuclear receptor in Th17 cells [24], and enhance extracellular signal-regulated kinase (ERK) 1 and two pathways that rise Foxp3^+^ expression [38], favoring Treg cells and reducing Th17 differentiation. 

RA ameliorates inflammation in a murine colitis model through IL-10 induction in T cells and Treg cells, which depends on toll-like receptor-2 binding [39]. In addition, vitamin A showed protective anti-inflammatory effects in a colitis model in rats, potentially through the preservation of mitochondrial activity [40]. Vitamin A also prevented colitis and colon cancer in a mouse model [41]. Moreover, Penny and colleagues showed that familial adenomatous polyposis in humans and mice resulted in reduced intestinal RA concentrations. Conversely, restoration of RA in vivo and in vitro led to increases in the number of Treg cells [42]. 

Arts and colleagues, while using monocytes from healthy volunteers, showed that vitamin A reduces cytokine production and this effect was mediated by histone methylation modifications [43], indicating epigenetic modulation as well. Whether the anti-inflammatory mechanisms of vitamin A supplementation are beneficial or not in the context of children exposed to malnutrition and enteric infections require further research. 

Liu and colleagues showed that vitamin A could improve intestinal immune response in the offspring of gestational vitamin A deficient rats when given during the early postnatal period. This was characterized by increased levels of secretory immunoglobulin A (sIgA) and CD8^+^ lymphocytes and dendritic cells. Of note, this effect was not observed with supplementation on later postnatal periods [44]. In addition, in utero maternal levels of RA control the size of secondary lymphocyte pools and the efficiency of immune responses in the offspring [37]. These observations have great relevance for further considerations of supplementing children with vitamin A deficiency, as the optimal time window for intervention is still a matter of debate. 

**Figure 1:**
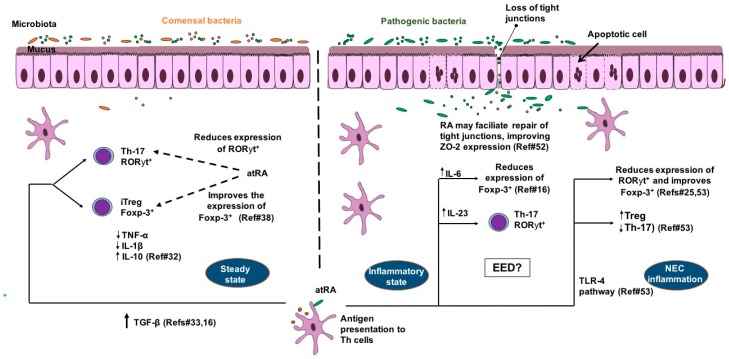
Model for the benefits of retinoic acid on intestinal barrier function in T-cell mediated inflammatory conditions such as environmental enteric dysfunction (EED) and neonatal necrotizing enterocolitis (NEC) in children. In steady state all-trans retinoic acid (RA) stimulates CD 103^+^ dendritic cells (DC) to produce more TGF-β, which favors Foxp-3^+^ T-reg differentiation rather than Th-17- retinoic acid receptor-related orphan receptor-gamma (ROR-γt^+^) cells. Foxp-3^+^ T-reg cells produce more anti-inflammatory cytokines (including IL-10) and less pro-inflammatory cytokines (TNF-α and IL-1β). In intestinal inflammation, RA is also important for Th17 ROR-γt^+^ for clearance of enteropathogens, with involvement of IL-6 and IL-23. In NEC inflammation, RA-TLR-4 pathway activation may reduce the expression of ROR-γt^+^ and favors Foxp-3^+^ differentiation rather than Th-17. RA may facilitate repair of tight junctions by improving ZO-2 expression during inflammatory conditions. Microbiota dysbiosis is a key factor in regulating RA effects under inflammatory conditions.

Altogether these studies provide important knowledge on how vitamin A may be essential for intestinal barrier function through immune modulatory responses, especially with anti-inflammatory effects, which may be helpful in chronic pro-inflammatory states, such as in children with EED. Figure 1 shows a model for the potential benefit of vitamin A-derived RA in this condition.

## 3. Interactions with Intestinal Epithelial Barrier, Enteric Pathogens and Microbiota

An important function of the intestinal mucosa is to act as a barrier between luminal contents and the underlying immune system. The physical epithelial barrier confers the property of selective permeability to the intestinal mucosa, which is tightly regulated in homeostasis and disrupted during disease. Intestinal permeability is a marker of intestinal barrier dysfunction [45]. The molecular structure of apical surface of the epithelium forms a single, continuous border as a result of the precise alignment of abutting cells from an apical to basal direction; the intercellular epithelial junctions are the tight junction (ZO; zonula occludens), adherens junction (zonula adherens), and the desmosome. The tight junction is the primarily regulator of paracellular permeability, maintained by the interaction between transmembrane claudins, occludins with ZO proteins to apical cytoskeletal proteins [46]. Although not yet fully understood, the influence of immunological components in regulating this epithelial protein complex has been accumulating. It has been recognized that intestinal inflammatory disorders (e.g., enteric infections, autoimmune diseases, among others) stimulate the production of TNF-α, a key etiologic mediator of intestinal barrier dysfunction [47]. Reduced intestinal mucosal Treg numbers have been associated with disrupted epithelial tight junctions [48].

Despite many studies have described the beneficial effects of vitamin A for regulation of immune response and intestinal epithelium (stimulating migration and proliferation) [49], little is known regarding how vitamin A directly modulates intestinal barrier function. Increased intestinal permeability is reported in the context of enteric infections and infant malnutrition (which can lead to vitamin A deficiency) associated with dysfunction of the intestinal epithelium barrier [50,51]. Recently, while using in vitro and pre-clinical studies, Li and colleagues showed that RA enhances ZO-2 expression, through TLR-4 activation to improve intestinal barrier function [52]. Furthermore, in a murine model of necrotizing enterocolitis (NEC), RA was able to reduce the severity of NEC by downregulating TLR-4-induction of IL-17 and to improve Tregs numbers and repopulation of LGR5^+^ intestinal stem cells [53,54]. TLR-4-mediated IL-17 activation causes loss of tight junctions in mice with NEC. These observations suggest that vitamin A should be further investigated in the context of EED in children from developing countries, in which the intestinal barrier function is compromised [55]. 

It is noteworthy that vitamin A regulates IL-22 responses, a major cytokine involved in intestinal barrier function homeostasis and repair and affecting epithelial tight junction’s claudin-2-related permeability [56]. RA induces IL-22 binding protein expression in dendritic cells, promoting intestinal homeostasis [57]. In addition, RA attenuates colon inflammation induced by dextran sodium sulfate treatment or *Citrobacter rodentium* infection with increased γδ T cells-derived IL-22 [58]. The interactions between intestinal immune and epithelial cells, which involve IL-22 and other mediators, are thus highly influenced by RA concentrations. 

Recently, the use of retinoids on specific enteric infections using both in vitro and in vivo approaches has been tested. Interestingly, the studies show differential effects depending on the type of pathogen. Cabrera and colleagues, using a murine model of Shiga toxin producing *E. coli* infection, found that vitamin A deficiency, while worsening intestinal damage during infection, improves survival. This unexpected finding may be related to the increased population of polymorphonuclear cells triggered by vitamin A, which enhances Shiga toxin effects [59]. In addition, McDaniel and colleagues showed that vitamin A-deficient mice infected with *Citrobacter rodentium*, a model for human *E. coli* infections, could become asymptomatically colonized if they do not succumb to premature lethality. In this model, RA supplementation improved pathogen clearance [60]. This study supports that vitamin A is important for defenses against *E. coli*-like pathogens, while reinforcing that vitamin A deficiency may contribute to asymptomatic colonization states. However, there is no data about direct antimicrobial effects of vitamin A molecules. Other studies have shown vitamin A deficiency impairs T and B responses to rotavirus vaccine in gnotobiotic piglet model [61,62].

While vitamin A modulation of the immune system is beneficial in some infections, it could be detrimental to others. Guerra-Perez and colleagues have described the potential worsening effects of vitamin A in the context of HIV infection. Dendritic cells supplemented with RA showed increased expression of the cell adhesion molecule MAdCAM-1, which was correlated with increased HIV replication. In addition, blocking MadCAM-1 partially inhibited HIV replication [63]. This biological mechanism may help to explain why vitamin A supplementation may not be beneficial in endemic HIV+ populations.

A major advance in understanding vitamin A transport during exposure to pathogens was made by the work of Derebe and colleagues [64]. They reported that serum amyloid A (SAA), induced during infections, functions as retinol binding proteins in both humans and mice. In an experimental murine model of enteric infection (*Salmonella typhimurium*), SAAs bound tightly to retinol in response to infection. Further, SAA-retinol binding sites were successfully identified by crystallographic analysis [64]. These observations add another link between immune system and vitamin A metabolism in the context of infections and suggest that more research should be focused on SAA [65]. 

Regarding the potential association of vitamin A effects with gut microbiota, little is known. Nevertheless, in an elegant study investigating the modulation of different micronutrients (vitamin A, folate, iron, and zinc) on gut microbiota, Hibberd and colleagues, while using gnotobiotic mice, found that vitamin A deficiency had the largest effect on microbial community and meta-transcriptome with increases in *Bacteroides vulgatus* in the context of vitamin A deprivation, results that could have important implications on bile acid metabolism [66]. These authors pointed out the need of more studies assessing vitamin A supplementation on gut microbiota of undernourished children. Another study showed that vitamin A exerts antiviral effects in vivo and in vitro against norovirus, and these effects were correlated with increases in *Lactobacillus* abundance in the gut [67]. Moreover, the effects of vitamin A on gut microbiota may explain other diseases. 

Xiao and colleagues have shown RA protection against necrotizing enterocolitis through intestinal microbiota and improved intestinal barrier function with increased protein expression of claudin-1, occludin, and ZO-1 [68]. Neonatal colitis has not received enough attention as a disease that may be impacted by vitamin A, a cause of mortality in preterm, undernourished newborns, and likely grossly under-diagnosed in low income societies where most births still occur at home. In addition, vitamin A supplementation has been shown to reduce infant mortality due to diarrhea in endemic areas of vitamin deficiency [69].

## 4. Clinical Evidence of Vitamin A for Enteric Diseases in Children: Adjusting Classical Paradigms and Facing New Challenges

According to WHO guidelines, vitamin A deficiency is defined as a severe public health problem in a setting where 20% of children aged 6–71 months have a serum retinol concentration <0.7 μmol/L [10,70]. It is estimated that 90 million pre-school children present with subclinical vitamin A deficiency [10]. Although further research is needed on biomarker development for vitamin A status, serum retinol is the most common laboratory measure [71]. 

Vitamin A deficiency and supplementation have been investigated more consistently in different settings in the past decades and these studies helped to guide major public health interventions in the developing world [72]. However, the general strong statement that vitamin A high dose supplementation should be prioritized for effectively reducing mortality of children aged six months to five years is now being questioned by some groups [73,74,75]. This debate should take into account the changing in the mortality rates of diarrheal diseases with sustained decline in the last decades, albeit with increasing morbidity of non-diarrheal EED, associated with enteric pathogens early in life, in the developing world [23,76]. This can be particularly important as this intervention is reported to cover more than 80% of total children population in developing countries [77]. 

A study by Fisker and colleagues evaluating the effects of vitamin A supplementation on children in a randomized double-blind trial at routine vaccination contacts in Guinea-Bissau showed no overall protective effect on mortality within six months of follow-up, however with an interaction between vitamin A supplementation and gender [78]. Gender differences have not been observed in either preschool aged children [79,80] or newborns [81,82] in trials in South Asia. Of note, the well-known benefit vitamin A supplementation had on child mortality reduction occurred in a time when vaccination programs could not reach high coverage and vitamin A deficiency rates were higher. Conversely, a recent pooled analysis of population-based surveys from the past two decades (1991–2013) reported that vitamin A deficiency is still prevalent in south Asia and sub-Saharan Africa, but has substantially declined in many countries from Southeast Asia, Oceania, Latin America, and the Caribbean. Mortality related to vitamin A deficiency in these settings has also declined throughout the past decades [74]. Nevertheless, vitamin A supplementation should consider the prevalence and burden of vitamin A deficiency within each country that could be uneven. In addition, poor diet diversification and heavy exposure to infections could explain why vitamin A deficiency was not reduced in some settings of south Asia and sub-Saharan Africa [74]. 

Overall, there is a great debate about the heterogeneity of vitamin A effects in clinical studies, and several hypotheses have been raised. It is accepted that the previous immunological state of the subjects should be considered, as infections can decrease vitamin A concentrations in the body as part of the acute-phase response. Thus, some authors suggest adjustment for these acute-phase markers, although there is no consensus on this matter [71,83]. There is also emerging evidence by genome-wide association studies supporting the existence of single nucleotide polymorphisms associated with vitamin A status, which suggest a contribution of genetic factors for the inter-individual variability in vitamin A status [84]. 

Recent large randomized clinical trials assessed the effects of neonatal vitamin A supplementation (children below six months of age) on mortality and morbidity outcomes. Two placebo-controlled studies that were performed in Tanzania and Ghana did not support vitamin A supplementation to young children [85,86]. However, trials in India and in Bangladesh found evidence of benefit for survival to 6 months of age [81,82,87]. The 10% reduction in mortality seen in the Mazumder’s India trial [87], although apparently modest, is epidemiologically important as a large proportion of deaths occur before supplementation is possible. 

Hamer and Keusch suggest that vitamin A supplementation policies should now focus on the reduction of deficiency itself rather than diarrhea morbidity or mortality as the outcome [88]. These observations corroborate the idea of rethinking vitamin A supplementation policies depending on the local epidemiology and risk of deficiency, as suggested by Stevens and colleagues (2015) [74]. Concerns related to potential adverse effects and correct dose are also raised by recent studies [89]. Furthermore, strategies targeting maternal vitamin A status as well as investments in social determinants should be made for addressing this issue [90]. Importantly, this crucial time-window in life may be characterized by heavy exposure to enteropathogens in children from developing countries, as recently shown by the Malnutrition and Enteric Diseases (MAL-ED) network cohort study [91].

Regarding studies on vitamin A effects in children older than 5 years, Thornton and colleagues assessed children aged 5–12 years from Colombia prospectively for one year. The study found that vitamin A deficiency (plasma retinol <10 μg/dL) was associated with an increased risk of diarrhea with vomiting and cough with fever. Consistently, these effects held after adjusting for sociodemographic characteristics and hemoglobin concentrations [92].

While the overall burden of vitamin A deficiency in public health is likely in part related to diarrhea and enteric infections, there are few studies that evaluated vitamin A deficiency or supplementation when comparing with the outcomes of specific enteric infections in children. In a randomized controlled trial in a Malaysian community with high endemicity of intestinal infections, a single high-dose of vitamin A supplementation was not able to protect against soil-transmitted helminth reinfections in children [93], and the authors suggested that more long-term interventions may be necessary to eliminate some parasites. While this may hold true, vitamin A may otherwise be associated with worsening infection outcomes. Interestingly, moderate to severe retinol deficiency was associated with reduced risk of incidence of EAEC and EPEC diarrhea in underweight and normal-weight children from urban Bangladesh [94].

Previously, vitamin A supplementation increased the duration of enteropathogenic *E. coli* infections in children aged 5–15 months from Mexico, possibly by decreasing IL-8 and monocyte chemoattractant protein-1 levels. On the other hand, vitamin A supplemented children showed shorter enterotoxigenic *E. coli* infections in association with fecal TNF-α and IL-6 concentrations [7]. Although not fully conclusive, altogether these findings corroborate that impairment of Th2 response (while favoring of Th1 profile) in the vitamin A deficiency state may harm the response against extracellular bacterial infections [92]. 

Another study with the same Mexican children revealed vitamin A modulates cytokine responses to norovirus infections, depending on the genogroup [7]. In addition, vitamin A supplementation reduced *Giardia* spp. infections in a double-blind, randomized placebo-controlled trial in children from northeastern Brazil [50]. Although it is well accepted that vitamin A differentially regulates the immune system and in turn can be beneficial and detrimental depending on the pathogen, more studies have to be done for understanding the complex immune response in which vitamin A plays. Overall these findings support the major role immune system plays in the response to vitamin A supplementation and could explain some inconsistent effects of vitamin A on diarrheal diseases outcomes, depending on the host immunologic profile. 

Recent studies have demonstrated the impact of EED for these populations, which is described as morphological and functional intestinal alterations in asymptomatic subjects living in highly contaminated environments [22,95]. This condition has been associated with impaired intestinal barrier function, leading to malnutrition, impaired intestinal absorption, vaccine response failure, and cognitive deficits in children [22]. Investigating how vitamin A interacts with EED is important, however, not many studies have addressed this issue properly. 

A recent study by Hossain and colleagues investigated children aged 6–24 months from Bangladesh and found plasma retinol was moderately associated with altered intestinal permeability [96]. Previously, low serum carotenoids, such as lutein, but not retinol, were suggested as good markers for the intestinal barrier function in children from an urban community in Fortaleza, Brazil. Further, serum retinol was correlated with acute-phase serum proteins [97]. However, vitamin A supplementation did not correlate with improved intestinal barrier function in children less than nine years old [50]. Of note, vitamin A status is also influenced by zinc levels, which is another major micronutrient that is associated with reduced risk of enteropathy and infections in children [71]. It is clear that more research assessing vitamin A interplay with intestinal microbiome and coinfections is needed in EED.

The changing epidemiology seen in child populations from developing countries must revise our understanding of vitamin A interventions in public health. While child mortality due to diarrhea is declining in many countries, we now recognize the detrimental consequences of EED for child’s health [98]. Elucidating how vitamin A deficiency is involved in EED is required. The recent progress of studies on the microbiome and its relationship with environmental and nutritional factors further support the idea that different populations may require different interventions. In this context, it is important that future clinical studies aim to characterize children’s vitamin A levels, immune response, infections status, and vitamin A interactions with other factors, such as intestinal microbiome, specific enteropathogens, and other micronutrients. These interactions might also contribute to the complexity of the current study findings.

## 5. Conclusions

Recent advances in our understanding of vitamin A signaling show that it is essential for intestinal function in children and sometimes has paradoxical effects. Both clinical and in vivo/in vitro vitamin A studies show complex effects on intestinal homeostasis. Importantly, vitamin A may lead to anti-inflammatory effects through lymphocyte modulation. Several factors may in turn modulate responses to vitamin A, such as the dose proposed for a given vitamin A status, the inflammatory/infectious conditions, and the gut microbiota. Moreover, it is clear that vitamin A plays differential roles in different enteric infections, depending on the type of immune response activated. Finally, further research using more integrative approaches, while considering inflammation/gut microbiome status and vitamin A measurements should be performed for better understanding the critical role vitamin A plays in enteropathy in children.

## References

[1] Gutierrez-Mazariegos J., Theodosiou M., Campo-Paysaa F., Schubert M. (2011). Vitamin A: A multifunctional tool for development. Semin. Cell. Dev. Biol..

[2] Kanungo J. (2017). Retinoic Acid Signaling in P19 Stem Cell Differentiation. Anticancer Agents Med. Chem..

[3] Sommer A. (2014). Preventing blindness and saving lives: The centenary of vitamin A. JAMA Ophthalmol..

[4] Paganelli A., Gnazzo V., Acosta H., Lopez S.L., Carrasco A.E. (2010). Glyphosate-based herbicides produce teratogenic effects on vertebrates by impairing retinoic acid signaling. Chem. Res. Toxicol..

[5] Robinson J.F., Verhoef A., Pennings J.L., Pronk T.E., Piersma A.H. (2012). A comparison of gene expression responses in rat whole embryo culture and in vivo: Time-dependent retinoic acid-induced teratogenic response. Toxicol. Sci..

[6] Green H.N., Mellanby E. (1928). Vitamin A as an anti-infective agent. Br. Med. J..

[7] Long K.Z., Santos J.I., Rosado J.L., Estrada-Garcia T., Haas M., Al M.A., DuPont H.L., Nanthakumar N.N. (2011). Vitamin A supplementation modifies the association between mucosal innate and adaptive immune responses and resolution of enteric pathogen infections. Am. J. Clin. Nutr..

[8] Mitter S.S., Oria R.B., Kvalsund M.P., Pamplona P., Joventino E.S., Mota R.M., Goncalves D.C., Patrick P.D., Guerrant R.L., Lima A.A. (2012). Apolipoprotein E4 influences growth and cognitive responses to micronutrient supplementation in shantytown children from northeast Brazil. Clinics (Sao Paulo).

[9] World Health Organization (WHO) (2005). Vitamin and Mineral Requirements in Human Nutrition.

[10] World Health Organization (WHO) (2009). Global Prevalence of Vitamin A Deficiency in Populations at Risk 1995–2005.

[11] Raverdeau M., Mills K.H. (2014). Modulation of T cell and innate immune responses by retinoic Acid. J. Immunol..

[12] Amengual J., Zhang N., Kemerer M., Maeda T., Palczewski K., Von L.J. (2014). STRA6 is critical for cellular vitamin A uptake and homeostasis. Hum. Mol. Genet..

[13] Blomhoff R., Blomhoff H.K. (2006). Overview of retinoid metabolism and function. J. Neurobiol..

[14] Rubin L.P., Ross A.C., Stephensen C.B., Bohn T., Tanumihardjo S.A. (2017). Metabolic Effects of Inflammation on Vitamin A and Carotenoids in Humans and Animal Models. Adv. Nutr..

[15] Mowat A.M., Agace W.W. (2014). Regional specialization within the intestinal immune system. Nat. Rev. Immunol..

[16] Omenetti S., Pizarro T.T. (2015). The Treg/Th17 axis: A dynamic balance regulated by the gut microbiome. Front. Immunol..

[17] Scott C.L., Aumeunier A.M., Mowat A.M. (2011). Intestinal CD103+ dendritic cells: Master regulators of tolerance?. Trends Immunol..

[18] Iwata M., Hirakiyama A., Eshima Y., Kagechika H., Kato C., Song S.Y. (2004). Retinoic acid imprints gut-homing specificity on T cells. Immunity.

[19] Mwanza-Lisulo M., Chomba M.S., Chama M., Besa E.C., Funjika E., Zyambo K., Banda R., Imikendu M., Sianongo S., Hancock R.E.W. (2018). Retinoic acid elicits a coordinated expression of gut homing markers on T lymphocytes of Zambian men receiving oral Vivotif, but not Rotarix, Dukoral or OPVERO vaccines. Vaccine.

[20] Sun C.M., Hall J.A., Blank R.B., Bouladoux N., Oukka M., Mora J.R., Belkaid Y. (2007). Small intestine lamina propria dendritic cells promote de novo generation of Foxp3 T reg cells via retinoic acid. J. Exp. Med..

[21] Bakdash G., Vogelpoel L.T., van Capel T.M., Kapsenberg M.L., de Jong E.C. (2015). Retinoic acid primes human dendritic cells to induce gut-homing, IL-10-producing regulatory T cells. Mucosal Immunol..

[22] Watanabe K., Petri W.A. (2016). Environmental enteropathy: Elusive but significant subclinical abnormalities in developing countries. EBioMedicine.

[23] Guerrant R.L., Leite A.M., Pinkerton R., Medeiros P.H., Cavalcante P.A., DeBoer M., Kosek M., Duggan C., Gewirtz A., Kagan J.C. (2016). Biomarkers of environmental enteropathy, inflammation, stunting, and impaired growth in children in northeast Brazil. PLoS ONE.

[24] Lu L., Lan Q., Li Z., Zhou X., Gu J., Li Q., Wang J., Chen M., Liu Y., Shen Y. (2014). Critical role of all-trans retinoic acid in stabilizing human natural regulatory T cells under inflammatory conditions. Proc. Natl. Acad. Sci. USA.

[25] Zhou X., Kong N., Wang J., Fan H., Zou H., Horwitz D., Brand D., Liu Z., Zheng S.G. (2010). Cutting edge: All-trans retinoic acid sustains the stability and function of natural regulatory T cells in an inflammatory milieu. J. Immunol..

[26] Ruemmele F.M., Garnier-Lengline H. (2013). Transforming growth factor and intestinal inflammation: The role of nutrition. Nestle. Nutr. Inst. Workshop Ser..

[27] Cha H.R., Chang S.Y., Chang J.H., Kim J.O., Yang J.Y., Kim C.H., Kweon M.N. (2010). Downregulation of Th17 cells in the small intestine by disruption of gut flora in the absence of retinoic acid. J. Immunol..

[28] Elias K.M., Laurence A., Davidson T.S., Stephens G., Kanno Y., Shevach E.M., O’Shea J.J. (2008). Retinoic acid inhibits Th17 polarization and enhances FoxP3 expression through a Stat-3/Stat-5 independent signaling pathway. Blood.

[29] Duester G. (2000). Families of retinoid dehydrogenases regulating vitamin A function: Production of visual pigment and retinoic acid. Eur. J. Biochem..

[30] Sandell L.L., Sanderson B.W., Moiseyev G., Johnson T., Mushegian A., Young K., Rey J.P., Ma J.X., Staehling-Hampton K., Trainor P.A. (2007). RDH10 is essential for synthesis of embryonic retinoic acid and is required for limb, craniofacial, and organ development. Genes Dev..

[31] Vicente-Suarez I., Larange A., Reardon C., Matho M., Feau S., Chodaczek G., Park Y., Obata Y., Gold R., Wang-Zhu Y. (2015). Unique lamina propria stromal cells imprint the functional phenotype of mucosal dendritic cells. Mucosal Immunol..

[32] Czarnewski P., Das S., Parigi S.M., Villablanca E.J. (2017). Retinoic acid and its role in modulating intestinal innate immunity. Nutrients.

[33] Coombes J.L., Siddiqui K.R., Arancibia-Carcamo C.V., Hall J., Sun C.M., Belkaid Y., Powrie F. (2007). A functionally specialized population of mucosal CD103+ DCs induces Foxp3+ regulatory T cells via a TGF-beta and retinoic acid-dependent mechanism. J. Exp. Med..

[34] Macpherson A.J., Slack E. (2007). The functional interactions of commensal bacteria with intestinal secretory IgA. Curr. Opin. Gastroenterol..

[35] Mora J.R., Iwata M., Eksteen B., Song S.Y., Junt T., Senman B., Otipoby K.L., Yokota A., Takeuchi H., Ricciardi-Castagnoli P. (2006). Generation of gut-homing IgA-secreting B cells by intestinal dendritic cells. Science.

[36] Bono M.R., Tejon G., Flores-Santibanez F., Fernandez D., Rosemblatt M., Sauma D. (2016). Retinoic acid as a modulator of T cell immunity. Nutrients.

[37] Van de Pavert S.A., Ferreira M., Domingues R.G., Ribeiro H., Molenaar R., Moreira-Santos L., Almeida F.F., Ibiza S., Barbosa I., Goverse G. (2014). Maternal retinoids control type 3 innate lymphoid cells and set the offspring immunity. Nature.

[38] Lu L., Ma J., Li Z., Lan Q., Chen M., Liu Y., Xia Z., Wang J., Han Y., Shi W. (2011). All-trans retinoic acid promotes TGF-beta-induced Tregs via histone modification but not DNA demethylation on Foxp3 gene locus. PLoS ONE.

[39] Nguyen V., Pearson K., Kim J.H., Kamdar K., DePaolo R.W. (2015). Retinoic acid can exacerbate T cell intrinsic TLR2 activation to promote tolerance. PLoS ONE.

[40] Reifen R., Levy E., Berkovich Z., Tirosh O. (2015). Vitamin A exerts its antiinflammatory activities in colitis through preservation of mitochondrial activity. Nutrition.

[41] Okayasu I., Hana K., Nemoto N., Yoshida T., Saegusa M., Yokota-Nakatsuma A., Song S.Y., Iwata M. (2016). Vitamin A inhibits development of dextran sulfate sodium-induced colitis and colon cancer in a mouse model. Biomed. Res. Int..

[42] Penny H.L., Prestwood T.R., Bhattacharya N., Sun F., Kenkel J.A., Davidson M.G., Shen L., Zuniga L.A., Seeley E.S., Pai R. (2016). Restoring retinoic acid attenuates intestinal inflammation and tumorigenesis in APCMin/+ mice. Cancer Immunol. Res..

[43] Arts R.J., Blok B.A., van C.R., Joosten L.A., Aaby P., Benn C.S., Netea M.G. (2015). Vitamin A induces inhibitory histone methylation modifications and down-regulates trained immunity in human monocytes. J. Leukoc. Biol..

[44] Liu X., Cui T., Li Y., Wang Y., Wang Q., Li X., Bi Y., Wei X., Liu L., Li T. (2014). Vitamin A supplementation in early life enhances the intestinal immune response of rats with gestational vitamin A deficiency by increasing the number of immune cells. PLoS ONE.

[45] Turner J.R. (2009). Intestinal mucosal barrier function in health and disease. Nat. Rev. Immunol..

[46] Yano T., Kanoh H., Tamura A., Tsukita S. (2017). Apical cytoskeletons and junctional complexes as a combined system in epithelial cell sheets. Ann. N. Y. Acad. Sci..

[47] Haines R.J., Beard R.S., Eitner R.A., Chen L., Wu M.H. (2016). TNFalpha/IFNgamma mediated intestinal epithelial barrier dysfunction is attenuated by MicroRNA-93 downregulation of PTK6 in mouse colonic epithelial cells. PLoS ONE.

[48] Mao J.W., Tang H.Y., Zhao T., Tan X.Y., Bi J., Wang B.Y., Wang Y.D. (2015). Intestinal mucosal barrier dysfunction participates in the progress of nonalcoholic fatty liver disease. Int. J. Clin. Exp. Pathol..

[49] Maciel A.A., Oria R.B., Braga-Neto M.B., Braga A.B., Carvalho E.B., Lucena H.B., Brito G.A., Guerrant R.L., Lima A.A. (2007). Role of retinol in protecting epithelial cell damage induced by Clostridium difficile toxin A. Toxicon.

[50] Lima A.A., Soares A.M., Lima N.L., Mota R.M., Maciel B.L., Kvalsund M.P., Barrett L.J., Fitzgerald R.P., Blaner W.S., Guerrant R.L. (2010). Effects of vitamin A supplementation on intestinal barrier function, growth, total parasitic, and specific *Giardia* spp. infections in Brazilian children: A prospective randomized, double-blind, placebo-controlled trial. J. Pediatr Gastroenterol. Nutr..

[51] Mondal D., Minak J., Alam M., Liu Y., Dai J., Korpe P., Liu L., Haque R., Petri W.A. (2012). Contribution of enteric infection, altered intestinal barrier function, and maternal malnutrition to infant malnutrition in Bangladesh. Clin. Infect. Dis..

[52] Li Y., Gao Y., Cui T., Yang T., Liu L., Li T., Chen J. (2017). Retinoic acid facilitates Toll-Like Receptor 4 expression to improve intestinal barrier function through retinoic acid receptor Beta. Cell. Physiol. Biochem..

[53] Egan C.E., Sodhi C.P., Good M., Lin J., Jia H., Yamaguchi Y., Lu P., Ma C., Branca M.F., Weyandt S. (2016). Toll-like receptor 4-mediated lymphocyte influx induces neonatal necrotizing enterocolitis. J. Clin. Investig..

[54] Nino D.F., Sodhi C.P., Egan C.E., Zhou Q., Lin J., Lu P., Yamaguchi Y., Jia H., Martin L.Y., Good M. (2017). Retinoic Acid improves incidence and severity of necrotizing enterocolitis by lymphocyte balance restitution and repopulation of LGR5+ intestinal stem cells. Shock.

[55] Crane R.J., Jones K.D., Berkley J.A. (2015). Environmental enteric dysfunction: An overview. Food Nutr. Bull..

[56] Wang Y., Mumm J.B., Herbst R., Kolbeck R., Wang Y. (2017). IL-22 Increases permeability of intestinal epithelial tight junctions by enhancing Claudin-2 expression. J. Immunol..

[57] Martin J.C., Beriou G., Heslan M., Chauvin C., Utriainen L., Aumeunier A., Scott C.L., Mowat A., Cerovic V., Houston S.A. (2014). Interleukin-22 binding protein (IL-22BP) is constitutively expressed by a subset of conventional dendritic cells and is strongly induced by retinoic acid. Mucosal Immunol..

[58] Mielke L.A., Jones S.A., Raverdeau M., Higgs R., Stefanska A., Groom J.R., Misiak A., Dungan L.S., Sutton C.E., Streubel G. (2013). Retinoic acid expression associates with enhanced IL-22 production by gammadelta T cells and innate lymphoid cells and attenuation of intestinal inflammation. J. Exp. Med..

[59] Cabrera G., Fernandez-Brando R.J., Abrey-Recalde M.J., Baschkier A., Pinto A., Goldstein J., Zotta E., Meiss R., Rivas M., Palermo M.S. (2014). Retinoid levels influence enterohemorrhagic Escherichia coli infection and Shiga toxin 2 susceptibility in mice. Infect. Immun..

[60] McDaniel K.L., Restori K.H., Dodds J.W., Kennett M.J., Ross A.C., Cantorna M.T. (2015). Vitamin A-deficient hosts become nonsymptomatic reservoirs of *Escherichia coli*-like enteric infections. Infect. Immun..

[61] Chattha K.S., Vlasova A.N., Kandasamy S., Esseili M.A., Siegismund C., Rajashekara G., Saif L.J. (2013). Probiotics and colostrum/milk differentially affect neonatal humoral immune responses to oral rotavirus vaccine. Vaccine.

[62] Vlasova A.N., Chattha K.S., Kandasamy S., Siegismund C.S., Saif L.J. (2013). Prenatally acquired vitamin A deficiency alters innate immune responses to human rotavirus in a gnotobiotic pig model. J. Immunol..

[63] Guerra-Perez N., Frank I., Veglia F., Aravantinou M., Goode D., Blanchard J.L., Gettie A., Robbiani M., Martinelli E. (2015). Retinoic acid imprints a mucosal-like phenotype on dendritic cells with an increased ability to fuel HIV-1 infection. J. Immunol..

[64] Derebe M.G., Zlatkov C.M., Gattu S., Ruhn K.A., Vaishnava S., Diehl G.E., MacMillan J.B., Williams N.S., Hooper L.V. (2014). Serum amyloid A is a retinol binding protein that transports retinol during bacterial infection. eLife.

[65] Esterhazy D., Mucida D. (2014). Serum amyloid A proteins take retinol for a ride. Trends Immunol..

[66] Hibberd M.C., Wu M., Rodionov D.A., Li X., Cheng J., Griffin N.W., Barratt M.J., Giannone R.J., Hettich R.L., Osterman A.L. (2017). The effects of micronutrient deficiencies on bacterial species from the human gut microbiota. Sci. Transl. Med..

[67] Lee H., Ko G. (2016). Antiviral effect of vitamin A on norovirus infection via modulation of the gut microbiome. Sci. Rep..

[68] Xiao S., Li Q., Hu K., He Y., Ai Q., Hu L., Yu J. (2018). Vitamin A and retinoic acid exhibit protective effects on necrotizing enterocolitis by regulating intestinal flora and enhancing the intestinal epithelial barrier. Arch. Med. Res..

[69] Tielsch J.M., Rahmathullah L., Thulasiraj R.D., Katz J., Coles C., Sheeladevi S., John R., Prakash K. (2007). Newborn vitamin A dosing reduces the case fatality but not incidence of common childhood morbidities in South India. J. Nutr..

[70] Black R.E., Victora C.G., Walker S.P., Bhutta Z.A., Christian P., de O.M., Ezzati M., Grantham-McGregor S., Katz J., Martorell R. (2013). Maternal and child undernutrition and overweight in low-income and middle-income countries. Lancet.

[71] Tanumihardjo S.A., Russell R.M., Stephensen C.B., Gannon B.M., Craft N.E., Haskell M.J., Lietz G., Schulze K., Raiten D.J. (2016). Biomarkers of nutrition for development (BOND)-vitamin A review. J. Nutr..

[72] Imdad A., Mayo-Wilson E., Herzer K., Bhutta Z.A. (2017). Vitamin A supplementation for preventing morbidity and mortality in children from six months to five years of age. Cochrane Database Syst. Rev..

[73] Benn C.S., Aaby P., Arts R.J., Jensen K.J., Netea M.G., Fisker A.B. (2015). An enigma: Why vitamin A supplementation does not always reduce mortality even though vitamin A deficiency is associated with increased mortality. Int. J. Epidemiol..

[74] Stevens G.A., Bennett J.E., Hennocq Q., Lu Y., De-Regil L.M., Rogers L., Danaei G., Li G., White R.A., Flaxman S.R. (2015). Trends and mortality effects of vitamin A deficiency in children in 138 low-income and middle-income countries between 1991 and 2013: A pooled analysis of population-based surveys. Lancet Glob. Health.

[75] Mason J.B., Benn C.S., Sachdev H., West K.P., Palmer A.C., Sommer A. (2018). Should universal distribution of high dose vitamin A to children cease?. BMJ.

[76] GBD Diarrhoeal Diseases Collaborators (2017). Estimates of global, regional, and national morbidity, mortality, and aetiologies of diarrhoeal diseases: A systematic analysis for the Global Burden of Disease Study 2015. Lancet Infect. Dis..

[77] Mason J., Greiner T., Shrimpton R., Sanders D., Yukich J. (2015). Vitamin A policies need rethinking. Int. J. Epidemiol..

[78] Fisker A.B., Bale C., Rodrigues A., Balde I., Fernandes M., Jorgensen M.J., Danneskiold-Samsoe N., Hornshoj L., Rasmussen J., Christensen E.D. (2014). High-dose vitamin A with vaccination after 6 months of age: A randomized trial. Pediatrics.

[79] Sommer A., Tarwotjo I., Djunaedi E., West K.P., Loeden A.A., Tilden R., Mele L. (1986). Impact of vitamin A supplementation on childhood mortality. A randomised controlled community trial. Lancet.

[80] West K.P., Pokhrel R.P., Katz J., LeClerq S.C., Khatry S.K., Shrestha S.R., Pradhan E.K., Tielsch J.M., Pandey M.R., Sommer A. (1991). Efficacy of vitamin A in reducing preschool child mortality in Nepal. Lancet.

[81] Rahmathullah L., Tielsch J.M., Thulasiraj R.D., Katz J., Coles C., Devi S., John R., Prakash K., Sadanand A.V., Edwin N. (2003). Impact of supplementing newborn infants with vitamin A on early infant mortality: Community based randomised trial in southern India. BMJ.

[82] Klemm R.D., Labrique A.B., Christian P., Rashid M., Shamim A.A., Katz J., Sommer A., West K.P. (2008). Newborn vitamin A supplementation reduced infant mortality in rural Bangladesh. Pediatrics.

[83] Bresnahan K.A., Tanumihardjo S.A. (2014). Undernutrition, the acute phase response to infection, and its effects on micronutrient status indicators. Adv. Nutr..

[84] Borel P., Desmarchelier C. (2017). Genetic variations associated with vitamin A status and vitamin A bioavailability. Nutrients.

[85] Edmond K.M., Newton S., Shannon C., O’Leary M., Hurt L., Thomas G., Amenga-Etego S., Tawiah-Agyemang C., Gram L., Hurt C.N. (2015). Effect of early neonatal vitamin A supplementation on mortality during infancy in Ghana (Neovita): A randomised, double-blind, placebo-controlled trial. Lancet.

[86] Masanja H., Smith E.R., Muhihi A., Briegleb C., Mshamu S., Ruben J., Noor R.A., Khudyakov P., Yoshida S., Martines J. (2015). Effect of neonatal vitamin A supplementation on mortality in infants in Tanzania (Neovita): A randomised, double-blind, placebo-controlled trial. Lancet.

[87] Mazumder S., Taneja S., Bhatia K., Yoshida S., Kaur J., Dube B., Toteja G.S., Bahl R., Fontaine O., Martines J. (2015). Efficacy of early neonatal supplementation with vitamin A to reduce mortality in infancy in Haryana, India (Neovita): A randomised, double-blind, placebo-controlled trial. Lancet.

[88] Hamer D.H., Keusch G.T. (2015). Vitamin A deficiency: Slow progress towards elimination. Lancet Glob. Health.

[89] Benn C.S., Diness B.R., Balde I., Rodrigues A., Lausch K.R., Martins C.L., Fisker A.B., Aaby P. (2014). Two different doses of supplemental vitamin A did not affect mortality of normal-birth-weight neonates in Guinea-Bissau in a randomized controlled trial. J. Nutr..

[90] Haider B.A., Bhutta Z.A. (2015). Neonatal vitamin A supplementation: Time to move on. Lancet.

[91] Platts-Mills J.A., Babji S., Bodhidatta L., Gratz J., Haque R., Havt A., McCormick B.J., McGrath M., Olortegui M.P., Samie A. (2015). Pathogen-specific burdens of community diarrhoea in developing countries: A multisite birth cohort study (MAL-ED). Lancet Glob. Health.

[92] Thornton K.A., Mora-Plazas M., Marin C., Villamor E. (2014). Vitamin A deficiency is associated with gastrointestinal and respiratory morbidity in school-age children. J. Nutr..

[93] Al-Mekhlafi H.M., Anuar T.S., Al-Zabedi E.M., Al-Maktari M.T., Mahdy M.A., Ahmed A., Sallam A.A., Abdullah W.A., Moktar N., Surin J. (2014). Does vitamin A supplementation protect schoolchildren from acquiring soil-transmitted helminthiasis? A randomized controlled trial. Parasit. Vectors.

[94] Ahmed A.M., Soares Magalhaes R.J., Long K.Z., Ahmed T., Alam M.A., Hossain M.I., Islam M.M., Mahfuz M., Mondal D., Haque R. (2016). Association of vitamin D status with incidence of enterotoxigenic, enteropathogenic and enteroaggregative Escherichia coli diarrhoea in children of urban Bangladesh. Trop. Med. Int. Health.

[95] Kosek M., Oberhelman R.A. (2007). Unraveling the contradictions of vitamin A and infectious diseases in children. J. Infect. Dis..

[96] Hossain M.I., Haque R., Mondal D., Mahfuz M., Ahmed A.S., Islam M.M., Guerrant R.L., Petri W.A., Ahmed T. (2016). Undernutrition, Vitamin A and Iron Deficiency Are Associated with Impaired Intestinal Mucosal Permeability in Young Bangladeshi Children Assessed by Lactulose/Mannitol Test. PLoS ONE.

[97] Vieira M.M., Paik J., Blaner W.S., Soares A.M., Mota R.M., Guerrant R.L., Lima A.A. (2008). Carotenoids, retinol, and intestinal barrier function in children from northeastern Brazil. J. Pediatr Gastroenterol. Nutr..

[98] Guerrant R.L., DeBoer M.D., Moore S.R., Scharf R.J., Lima A.A. (2013). The impoverished gut—A triple burden of diarrhoea, stunting and chronic disease. Nat. Rev. Gastroenterol. Hepatol..

